# Editorial: Mechanisms of biofilm development and antibiofilm strategies

**DOI:** 10.3389/fmicb.2023.1190611

**Published:** 2023-04-04

**Authors:** Liangyue Pang, Huancai Lin, Fang Yang, Dongmei Deng

**Affiliations:** ^1^Hospital of Stomatology, Sun Yat-sen University, Guangzhou, Guangdong, China; ^2^Guangdong Provincial Key Laboratory of Stomatology, Sun Yat-sen University, Guangzhou, Guangdong, China; ^3^Qingdao Hospital, University of Health and Rehabilitation Sciences, Qingdao, Shandong, China; ^4^Department of Preventive Dentistry, Academic Center for Dentistry Amsterdam (ACTA), University of Amsterdam and VU University Amsterdam, Amsterdam, Netherlands

**Keywords:** biofilm, mechanism, antibiofilm, antimicrobial agents, antibiofilm strategies

Biofilms refer to the surface-attached groups of microbial cells embedded in an extracellular matrix. Biofilms' development is a complex dynamic process, including five stages: initial attachment, EPS production leading to “irreversible” attachment, early development of biofilm architecture, maturation of biofilm architecture and dispersion of single cells (Tim, 2015). It is estimated that biofilms are related to 65–80% of infectious diseases. Understanding the biological mechanisms associated with the biofilm development process is of great significance for the development of novel antibiofilm agents. It has been known that microbes in biofilms are less susceptible to antimicrobial agents than their planktonic counterparts, and as a result, biofilm-related diseases are extremely difficult to prevent and cure (Wang et al., 2022). Most antibiofilm agents available till date have been antimicrobial agents with bactericidal effects, which are ineffective for treating biofilm-related infections and can lead to microbial resistance when used for a long time (Sun et al., 2013). Hence, it is critically important to design or screen novel antibiofilm agents that can effectively prevent biofilm formation or eradicate existing biofilm. We are honored to serve as Guest Editors, aiming to gather contributions to the Frontiers Research Topic on “Mechanisms of Biofilm Development and Antibiofilm Strategies”. We have compiled 15 papers which present state-of-the-art knowledge on the Research Topic, covering various bacterial species, namely *Pseudomonas aeruginosa, Acinetobacter baumannii, Corynebacterium matruchotii, Staphylococcus aureus, Streptococcus mutans*, and *Candida albicans*. The studies published in this Research Topic can be categorized into two groups: (1) the mechanisms underlying biofilms' development stages and (2) novel strategies to prevent biofilm formation or eradicate existing biofilm.

Extracellular DNA (eDNA) is a critical component in the extracellular matrix (ECM) of bacterial biofilms (Devaraj et al., 2019). Wang Y. et al. used the *Myxococcus xanthus* biofilm model to investigate the mechanisms underlying how eDNA integrated into the ECM through potential macromolecular interactions. Their results showed that eDNA was able to combine with *M. xanthus* EPS to form a macromolecular conjugate. During the biofilm formation process, the eDNA-EPS complex not only facilitated the initial cell adhesion and subsequent establishment of ECM architecture, but also renderd cells within biofilms stress resistances that are relevant to the survival of *M. xanthus* in hostile environments. Furthermore, the EPS protected the conjugated DNA from degradation by nucleic acid hydrolases, which led to the continuous and stable existence of eDNA in the native ECM of *M. xanthus* biofilms.

Biofilm formation has been found to be closely related to the drug resistance of *Acinetobacter baumannii*. Yang et al. focus on a new gene labeled with a locus tag of BIT33_RS14560 in the NCBI database. This new gene belongs to the Major Facilitator superfamily, some of which have been confirmed to be closely related to biofilm formation and drug resistance. They demonstrated that when BIT33_RS14560 was overexpressed in *A. baumannii*, the biofilm formation capacity and virulence were both enhanced, and they also presented RNA sequencing evidence to elucidate possible mechanisms for these changes.

Quorum sensing (QS), as the main signaling mechanism bacteria use for cell-to-cell communication, plays a key role in biofilm formation (Zhou et al., 2020). Wang N. et al. demonstrated that the QS regulatory protein PdeR promoted biofilm formation in *P. denitrificans*. They also revealed the underlying regulatory mechanism of PdeR during biofilm development: PdeR mainly promoted the intracellular degradation of amino acids and fatty acids, as well as siderophore biosynthesis and transportation, thus providing cells with enough energy and iron to form a thicker biofilm. This finding contributes to our understanding of QS regulation in biofilm development. A new antibacterial strategy based on inhibiting bacterial quorum sensing has emerged as a promising method of attenuating bacterial pathogenicity and preventing bacterial resistance to antibiotics. Bai et al. examined Echinatin (Ech) with high-efficiency anti-QS and verified the significantly synergistically increased antibacterial activity in overcoming the antibiotic resistance of *E. coli*, suggesting the potent anti-QS and novel antibacterial synergist candidate of Ech for treating *E. coli* infections.

*Corynebacterium matruchotii* is a gram-positive calcifying bacterium which presents in dental plaque on the tooth surface and serves as one of the most predominant bacterial species at the site. Li et al. reviewed the role of *C. matruchotii* in supragingival plaque based on biofilm structure, microbial interactions, and potential connections with oral diseases. The coexistence of *S. mutans* and *C. albicans* is closely related to the progression of early childhood caries, and exopolysaccharides play important roles in the cross-kingdom interaction between *S. mutans* and *C. albicans*. Lu et al. constructed the *C. albicans DCR1* low-expressing and over-expressing strains and co-cultured them with *S. mutans* wild type or *rnc*-mutant strains to explore the roles of *rnc* and *DCR1* in the modulation of dual-species biofilms of *S. mutans* and *C. albicans*. Their result indicating that the *DCR1* gene in *C. albicans* regulated the fungal yeast-to-hyphae transition, spatial structure, acid production and EPS/microorganism volume ratio of biofilms. While *rnc* gene in *S. mutans* prominently contributed to biofilm formation by increasing biofilm extracellular polysaccharide synthesis and *C. albicans* virulence, resulting in increased biomass, biofilm roughness, and acid production of the dual-species biofilms, which facilitated the assembly of a cariogenic cross-kingdom biofilm and the generation of an intensive acidic milieu.

Bacteria in the biofilm state are more tolerant to various antibiotics and, thus, are more difficult to control than bacteria in the planktonic state, and this sharply limits the application of existing antimicrobial therapies. Dispersion of biofilms is a promising avenue for the treatment of biofilm-associated diseases. To some extent, dispersion and eradication of mature biofilms are very important for biofilm control. Inducing dispersion would be a gentle and specific approach that would not influence the development of dysbiosis or the balance of the oral microbiome (Lin et al., 2022). Furthermore, the bacteria become much more sensitive to antimicrobial agents as they separate from the biofilm. Chen Y. et al. summarized strategies for the dispersion of cariogenic biofilms, including biofilm environment, signaling pathways, biological therapies, and nanovehicle-based adjuvant strategies. These strategies may provide great opportunities for the clinical treatment of dental diseases in the future.

*Pseudomonas aeruginosa* is one of the top three major pathogens implicated in human opportunistic infections and a common cause of clinically persistent infections. *P. aeruginosa* biofilm formation results in multiple antibiotic resistance, posing a significant challenge to conventional antibiotic therapeutic approaches. Yin et al. briefly introduced the process and regulation of *P. aeruginosa* biofilm formation and reviewed several traditional methods for biofilm treatment as well as current innovative treatment technologies with significant curative effects, providing new directions for the treatment of *P. aeruginosa* biofilm infection. Wang M. et al. investigated the antibiofilm property and antibiofilm pathway of a new antimicrobial peptide, named PEW300, against *P. aeruginosa*. Their results showed that PEW300 exhibited strong antibiofilm activity against *P. aeruginosa*. PEW300 dispersed biofilm preferentially by degrading extracellular DNA and adopted multiple action modes to cause cell death. In addition, PEW300 could dramatically reduce the virulence of *P. aeruginosa* by down-regulating the expression of virulence genes. Their results suggested that PEW300 has good potential to be an efficient antibacterial agent to combat *P. aeruginosa* biofilm.

Bacterial biofilm is an important mechanism mediating the antibiotic resistance of methicillin-resistant *S. aureus* (MRSA) as well. Using small molecules to enhance the efficacy of existing antibiotics is an economical and practical strategy to cope with biofilm-induced resistance. Chen R. et al. investigated the synergies of two small molecules, isobavachalcone and curcumin, both with anti-osteoporosis, anti-inflammation, and anti-bacteria characteristics, and demonstrated that the combination of these two molecules could enhance the susceptibility of MRSA to gentamicin, thus promoting the eradication of MRSA biofilm. When administered as a cocktail *in vivo*, they could significantly modify local inflammation in orthopedic implant-related infection and protect bone microstructure. Biofilms are recalcitrant to antibiotic treatment due to multiple tolerance mechanisms (phenotypic resistance) (Ciofu et al., 2017). Therefore, effective biofilm-targeting compounds are currently highly sought after. Long et al. found Elasnin as a potent and safe biofilm eradicator against MRSA which destroyed their biofilm matrix, reduced virulence, and increased their sensitivity to β-lactam antibiotics. Elasnin's mode of action was also elucidated, which highlighted the key genes that govern this process and the crucial role of *sarZ* during elasnin-induced biofilm eradication, pointing out the potential role of *sarZ* in regulating staphylococcal biofilm development. This study provides new strategies against the eradication of MRSA biofilms and new insights into the molecular targets for biofilm eradication in MRSA.

Now, more and more documents have focused on antimicrobial peptides (AMPs) as a substitute candidate for conventional antimicrobial agents against Multidrug-resistant (MDR) and biofilm-associated infections. Mirzaei et al. evaluated melittin's antibacterial and antibiofilm activity alone and/or in combination with gentamicin, ciprofloxacin, rifampin, and vancomycin on biofilm-forming MDR-*P. aeruginosa* and MDR-MRSA strains. Their results showed that melittin alone was effective against the strong biofilm of MDR pathogens and also offered sound synergistic effects with antibiotics without cytotoxicity, suggesting combining melittin and antibiotics can be a potential candidate for further evaluation of *in vivo* infections by MDR pathogens. Villanueva et al. attempted optimizing the antibiofilm potency of different commercially available tannins against bacteria by modifying their chemical structure with different derivatizations. Their results showed distinct correlations between certain chemical qualities of the tannins and their antibiofilm activity and spectrum. The authors suggested that the spectrum and the antibiofilm potency of the tannins could be modulated by the applied chemical modifications.

Natural products are promising medicines against the over-growth of oral pathogens in biofilms due to their excellent antibiofilm effects, abundant sources, relatively low cost, and safety. Chi et al. summarized the antibiofilm effects and mechanisms of natural products against mono- or multi-species biofilms, targeting the different stages of biofilm development, including adhesion, cell proliferation, maturation, and dispersion, as well as the combinational advantages with other strategies to enhance the antibiofilm properties of natural products, providing a new direction for antibiofilm agents. EPS plays a role in altering microbial behavior and virulence and can also enhance bacterial drug resistance (Gupta et al., 2016; Liu et al., 2016); targeting EPS may be an effective breakthrough point for removing or controlling biofilms.  established a periodontitis-related microbial multi-species biofilm containing the EPS encapsulating bacterial group and demonstrated that bovine trypsin could destroy the biofilm structure, dispersed the biofilm and bacteria, and significantly reduced the amount of EPS and biomass.

Altogether, this Research Topic outlines the mechanisms underlying biofilms' development stages and the novel strategies to prevent biofilm formation or eradicate existing biofilm. The information availed in the articles will advance our understanding of the mechanisms of biofilm development and antibiofilm strategies. The data presented in these articles will contribute to providing new directions for antibiofilm agents.

## Author contributions

All authors listed have made a substantial, direct, and intellectual contribution to the work and approved it for publication.
